# Metacognitive Differences in Amnestic Mild Cognitive Impairment and Healthy Cognition: A Cross-Sectional Study Employing Online Measures

**DOI:** 10.3390/jintelligence11090184

**Published:** 2023-09-19

**Authors:** Grigoria Bampa, Magdalini Tsolaki, Despina Moraitou, Panagiota Metallidou, Elvira Masoura, Maria Mintziviri, Konstantinos Paparis, Dorothea Tsourou, Georgia Papantoniou, Maria Sofologi, Vasileios Papaliagkas, Georgios Kougioumtzis, Efthymios Papatzikis

**Affiliations:** 1 Laboratory of Psychology, Department of Cognition, Brain and Behavior, School of Psychology, Aristotle University of Thessaloniki, 54124 Thessaloniki, Greece; demorait@psy.auth.gr (D.M.); pmetall@psy.auth.gr (P.M.); emasoura@psy.auth.gr (E.M.); 2 Laboratory of Neurodegenerative Diseases, Center of Interdisciplinary Research and Innovation (CIRI–AUTH), Balcan Center, Buildings A & B, 10th km Thessaloniki-Thermi, 54124 Thessaloniki, Greece; tsolakim1@gmail.com; 3 Greek Association of Alzheimer’s Disease and Related Disorders (GAADRD), 54643 Thessaloniki, Greece; 4 School of Psychology, Aristotle University of Thessaloniki, 54124 Thessaloniki, Greece; 5 Laboratory of Psychology, Department of Early Childhood Education, School of Education, University of Ioannina, 45110 Ioannina, Greece; gpapanto@uoi.gr (G.P.); m.sofologi@uoi.gr (M.S.); 6 Institute of Humanities and Social Sciences, University Research Centre of Ioannina (URCI), 45110 Ioannina, Greece; 7 Department of Biomedical Sciences, School of Health Sciences, International Hellenic University, 57400 Thessaloniki, Greece; vpapaliagkas@gmail.com; 8 Department of Turkish and Modern Asian Studies, National and Kapodistrian University of Athens, 15772 Athens, Greece; gkougioum@ppp.uoa.gr; 9 Department of Psychology, Neapolis University Pafos, Pafos 8042, Cyprus; 10 Department of Early Childhood Education and Care, Oslo Metropolitan University, 0167 Oslo, Norway; 11 College of Medicine and Health Sciences, Khalifa University, Abu Dhabi 127788, United Arab Emirates

**Keywords:** metacognitive control, metacognitive monitoring, feeling of confidence, mild cognitive impairment, executive functions, memory

## Abstract

This study aimed to examine metacognitive abilities in individuals diagnosed with amnestic mild cognitive impairment (aMCI) by using online metacognitive measures during cognitive tasks. A total of 100 participants were enrolled, all aged 50 or older (mean age = 61.98; SD = 6.27), and with a minimum of six years of education (mean = 14.95; SD = 2.94). The sample included 50 individuals with aMCI (34 females) and 50 healthy controls (HC) (33 females). Both groups underwent metacognitive versions of memory tasks (Doors and People) and executive functions tasks (Wisconsin Card Sorting Test). Metacognition was assessed through confidence ratings given after each answer (referred to as metacognitive monitoring) and the accuracy of the participants’ decisions to include or exclude answers from their final scores (known as metacognitive control). The results showed that although individuals with aMCI were aware of their cognitive limitations—evidenced by their lower confidence ratings across all tasks—they still exhibited overconfidence relative to their actual performance. Moreover, they included a greater number of incorrect answers in their final scores compared to the healthy control group. These findings suggest that while individuals with aMCI retain some level of awareness, their self-evaluations appear to lack precision. This observation was consistent across both types of cognitive tasks. The results underscore the need for additional research to better understand metacognition in MCI as well as the interplay between metacognitive monitoring and control.

## 1. Introduction

Mild cognitive impairment (MCI) affects a significant portion of the aging population. It is characterized by a decline in one or more cognitive domains that is not severe enough to interfere with an individual’s independence in daily activities (Petersen 2004). MCI is usually divided into amnestic (aMCI) and nonamnestic (naMCI) subtypes, depending on whether the primary cognitive deficits are in memory or (an)other cognitive domain(s), respectively (Petersen 2004; Petersen et al. 2014). Research has shown that different etiology and progression pathways characterize each subtype, with aMCI most likely representing an early manifestation of Alzheimer’s disease (AD). In contrast, naMCI represents a prodromal stage of non-AD dementias (Petersen et al. 2014). Although individuals with MCI can function self-sufficiently in most everyday life activities, this condition significantly impacts the quality of their lives (Stites et al. 2018). Therefore, it is crucial to understand the cognitive changes that occur in this population and develop interventions that can improve cognitive and daily functioning.

Metacognition is critical in maintaining cognitive abilities (Hertzog and Dunlosky 2011). It is a higher-level cognitive system comprising three key components: metacognitive knowledge, metacognitive monitoring, and metacognitive control (Flavell 1979; Dunlosky and Metcalfe 2008; Nelson and Narens 1990). Metacognitive knowledge encompasses an individual’s comprehension of general cognitive principles and their beliefs about their cognitive abilities (Schraw and Moshman 1995). While engaging in cognitive tasks, individuals experience metacognitive phenomena, such as task-related feelings and judgments (Efklides 2001), which offer “online” metacognitive knowledge that supports metacognitive monitoring processes (Efklides 2006). Examples of metacognitive experiences include feelings of confidence (FOCs, retrospective judgments for the correctness of a given response), judgments of learning (JOLs, prospective judgments of the likelihood of recalling/recognizing a given stimulus), and feelings of knowing (FOKs, a subjective evaluation, during retrieval, of the likelihood that one will recognize an item that they are currently unable to retrieve). Metacognitive monitoring describes the ability to evaluate ongoing cognitive processes, while metacognitive control describes the ability to regulate cognitive behavior toward achieving desired cognitive goals (Nelson and Narens 1990; Flavell 1979). All metacognitive components are interrelated and work together to orchestrate cognitive processes (Hertzog and Dunlosky 2011; Dunlosky and Metcalfe 2008; Efklides 2011). 

In assessing metacognition, researchers can utilize both offline and online measures. Offline measures, such as questionnaires, yield information about individuals’ metacognitive knowledge concerning their general cognitive status, insight into cognitive changes, satisfaction with cognitive skills, and strategy usage. In contrast, online measures, like judgments and estimations during cognitive tasks, evaluate monitoring and control processes for specific tasks. Metacognitive judgments and estimations can be gathered at the item level or for overall performance (global post-/predictions). Notably, the two approaches are not interchangeable and evaluate different aspects of metacognition (Clare et al. 2013). 

Numerous studies examining the significance of metacognition in later adulthood have demonstrated that older adults often overestimate their performance of cognitive tasks, as reflected by JOLs (Cauvin et al. 2019; McGillivray and Castel 2011; Siegel and Castel 2019; Hansson et al. 2008) and confidence ratings (Palmer et al. 2014; Hansson et al. 2008; Hertzog et al. 2021; Dodson et al. 2007). This overconfidence can be mitigated with task-related experience and feedback (McGillivray and Castel 2011; Siegel and Castel 2019). However, older adults still tend to remain more confident in their overall predictions than younger adults. Metacognitive accuracy in this age group appears to vary across different tasks, being impaired in recall, recognition, and visual perception tasks (Dodson et al. 2007; Hertzog et al. 2021; McGillivray and Castel 2011; Palmer et al. 2014; Perrotin et al. 2006; Siegel and Castel 2019) but often intact or even improved in tasks related to general knowledge (Dodson et al. 2007; Morson et al. 2015). Furthermore, a distinction exists in the monitoring accuracy between episodic and semantic memory in older adults, with semantic memory largely unaffected (Morson et al. 2015; Souchay et al. 2007; Perrotin et al. 2006). Finally, when controlling for cognitive performance, age-related disparities in metacognitive accuracy often decrease or even disappear, suggesting that differences in metacognition between age groups may primarily be driven by underlying cognitive abilities (Zakrzewski et al. 2021; Hertzog et al. 2021; Hansson et al. 2008).

There are also mixed findings regarding metacognitive control in older adults. Some studies have shown age-related deficits in study time allocation and decision-making (Tullis and Benjamin 2012; Froger et al. 2011), while others have reported that older adults can effectively recall information and strategically allocate study time based on its assigned value (Li et al. 2018; Siegel and Castel 2019; Murphy et al. 2023). McGillivray (2021) provides a comprehensive overview of the findings on metacognition in older adulthood, highlighting that factors such as motivation, personal interest, and emotional valence play an essential role in older adults’ metacognitive monitoring and control skills. In other words, these findings indicate that older adults’ control processes prioritize positive and personally valuable stimuli. 

Some studies have explored metacognition in individuals with MCI using offline measures such as self-perceptions of cognitive abilities, strategic utilization, and the prevalence of cognitive biases (Clare et al. 2013; Galeone et al. 2011; Vogel et al. 2004; Lin et al. 2020; Tomaszewski Farias et al. 2018). However, examining metacognition during active engagement with cognitive tasks has received comparatively less attention. The available findings, which are still limited in this area, present heterogeneity depending on several factors, such as the MCI subtype, the severity of cognitive deterioration, the type of metacognitive measure that was tested, and the applied cognitive tasks (for a review, see Piras et al. 2016).

Specifically, several studies employing online measures for overall performance have indicated that individuals with MCI appear to possess a fair degree of accuracy when it comes to evaluating their performance of a given task, as demonstrated by global prediction and postdiction measures (Seelye et al. 2010; Clare et al. 2013; Chudoba and Schmitter-Edgecombe 2020). These studies utilized memory recall tasks, with one study (Chudoba and Schmitter-Edgecombe 2020) also employing a functional capacity task (the Day-Out Task, DOT), which is a naturalistic task assessing everyday functioning (Schmitter-Edgecombe et al. 2012). In contrast to these results, Ryals et al. (2019) employed a recognition memory task for verbal and visual stimuli. They found that MCI participants underestimated their performance, as evidenced by their global predictions and postdictions. This highlights a domain-specific variation in metacognitive monitoring abilities in MCI individuals, who can accurately monitor their performance in specific tasks (memory recall and functional capacity tasks) but struggle in memory recognition tasks. This may stem from the inherent distinctions between recognition and recall processes (Eichenbaum et al. 2007). Recognition largely depends on automatic, familiarity-based processes, while recall involves more effortful, strategic retrieval processes (Unsworth and Spillers 2010). Consequently, metacognitive indicators may have a stronger connection to recall tasks, where the integration of cognitive and metacognitive components is more pronounced (Nelson and Narens 1990).

The findings in studies employing online measures of metacognition at the item-by-item level show considerable heterogeneity, too (Perrotin et al. 2007; Akhtar et al. 2006; Ryals et al. 2019; Chi et al. 2022; Anderson and Schmitter-Edgecombe 2010; Pennington et al. 2021). Studies examining metacognitive monitoring accuracy during memory retrieval, as indicated by FOK judgments (Anderson and Schmitter-Edgecombe 2010; Perrotin et al. 2007; Ryals et al. 2019; Chi et al. 2022) and FOC ratings after a given response (Chi et al. 2022; Ryals et al. 2019), have found deficits in aMCI participants. However, when measuring metamemory monitoring during encoding processes using JOLs (Ryals et al. 2019; Akhtar et al. 2006), aMCI participants performed equally well compared with healthy older adults. In contrast, naMCI participants exhibited deficits in metamemory monitoring when assessed with JOLs. Interestingly, variations in monitoring accuracy have been identified across distinct memory modalities. Notably, Ryals et al. (2019) found that individuals with aMCI exhibited more pronounced inaccuracies in memory awareness, as measured by FOK judgments, for verbal stimuli compared to visual stimuli. In addition, a recent study (Chi et al. 2022) examined differences in monitoring accuracy, as represented by FOC estimations, among healthy older adults, individuals with subjective cognitive impairment (SCI), and those with aMCI and naMCI, on a semantic memory task. The results demonstrated that both MCI groups had significantly poorer accuracy between their confidence judgments and performance than healthy controls and those with SCI. In their recent study, Pennington et al. (2021) studied metacognition, recruiting older adults with MCI, functional cognitive disorder (FCD), and healthy controls (HC) and examining their mean confidence and metacognitive efficacy in memory (verbal recognition) and visuospatial perception tasks. The MCI and FCD groups reported lower, albeit statistically insignificant, mean confidence in both tasks than the HC group. Also, significant differences were observed in metacognitive efficacy among groups in either task. The authors inferred that the absence of detected metacognitive deficits in the MCI group might be due to being in the early stages of MCI. However, a significant difference was detected in task modalities, with both HC and FCD groups exhibiting superior metacognitive efficacy in memory tasks over perceptual tasks, indicating a domain specificity in metacognition. In contrast, the MCI group did not show differentiation in metacognitive accuracy between the two tasks.

In conclusion, the relationship between metacognition and MCI is multifaceted, yielding varied findings across studies. Individuals with MCI often demonstrate accurate performance evaluation using global prediction and postdiction measures. However, this accuracy may depend on the type of cognitive task employed. Furthermore, online measures of metacognition reveal varying degrees of metacognitive monitoring deficits in individuals with MCI, with distinctions arising based on the type of memory system assessed and the cognitive process stage. Notably, there is a lack of evidence regarding metacognitive control in MCI, which warrants further investigation. These inconsistencies underscore the complex nature of metacognitive mechanisms and highlight the need for further investigation. McWilliams and colleagues (2023) recently addressed some of these issues, demonstrating an age-related decline in global and local confidence ratings and sustained metacognitive efficiency. These results were detected in memory and perceptual tasks, advocating the idea of domain generality in metacognitive aging, specifically within the context of normal aging rather than processes of neurodegeneration.

Hence, further research is necessary to elucidate the details of metacognitive alterations in MCI and its subtypes and to provide a better understanding of this condition and the development of more targeted interventions.

### Aim and Hypothesis of the Present Study

The objective of this study was to expand upon the current understanding of metacognition in aMCI. While existing research has mainly emphasized metamemory, exploring metacognition within various cognitive domains is vital since metacognitive aging might be domain-specific rather than general. Furthermore, to the best of our knowledge, no previous studies have probed into metacognitive control in aMCI, a point of interest in our study as reflected by the participants’ decisions to include or exclude responses from their final score and their accuracy to discriminate between right/wrong responses. We aimed to assess metacognition in people with aMCI characterized by multiple domain deficits—a condition potentially progressing to AD dementia—by utilizing online metacognitive measures in two cognitive tasks: a memory task with recall and recognition components and an executive functions task.

Based on the theoretical framework and available data outlined above, the following hypotheses were formulated:

We expected that participants with aMCI would perform worse than cognitively healthy (HC) older adults in both cognitive tasks, indicating inferior cognitive performance in the aMCI group (Hypothesis 1). 

With respect to metacognitive monitoring, we anticipated differences between the two groups. More specifically, we hypothesized that individuals with aMCI would express lower confidence levels than HC individuals (Hypothesis 2a), but they would show poorer calibration in relation to their performance (Hypothesis 2b).

To our knowledge, no prior study has evaluated metacognitive control in MCI. However, drawing from past research demonstrating deficits in metacognitive accuracy in MCI, we conjectured that participants in the aMCI group would display reduced precision in their decisions to volunteer correct or incorrect responses compared to the participants in the HC group (Hypothesis 3).

## 2. Materials and Methods

### 2.1. Design

This study employed a cross-sectional design comparing two distinct groups: (a) cognitively healthy older adults (HC ≥ 50 years) and (b) people with aMCI. By collecting data at a single point in time, the present study aimed to investigate potential differences between the two groups regarding cognitive and, primarily, metacognitive measures.

### 2.2. Participants

Initially, a power analysis was conducted using G*Power (Faul et al. 2007) for F-test: MANOVA Global Effects. The results suggested a sample size of at least 80 participants to achieve a power of 0.80. In total, 120 individuals were enlisted and assessed for their cognitive health. Among these, one individual was diagnosed with subjective mild cognitive impairment (SCI), another with naMCI, and two others were identified with cardiac issues. Consequently, these four participants were excluded from our study. From the remaining 116 participants, 54 were found to be cognitively healthy. Out of them, 51 agreed to participate in this study, and 1 participant began the first testing session but stopped for personal reasons. Of the 62 participants diagnosed with aMCI-md, 50 agreed to participate in this study.

Consequently, this study included 100 participants, 33 men and 67 women, with a mean age of 61.98 (SD = 6.27) years and a mean education of 14.95 (SD = 2.94) years. To participate in this study, individuals were required to be native Greek speakers, be over age 50, and have a minimum of six years of education. This study explicitly enlisted participants exhibiting the amnestic subtype of MCI characterized by multiple deficits. Thus, if the individual’s memory and one or more additional cognitive areas as evaluated through neuropsychological tests were significantly below the norm for their age (i.e., 1.5 standard deviations), they would then be classified as aMCI-md (Winblad et al. 2004). Given the global rise of AD dementia and considering that aMCI often represents a prodromal stage of AD, the risk of future AD dementia becomes even more significant in multidomain aMCI (Petersen et al. 2014), and this is why we focused on this specific subtype. All participants underwent an extended neuropsychological assessment to discriminate between those with healthy cognitive status and those with aMCI (multiple deficits) in accordance with Petersen’s diagnostic criteria (Petersen et al. 2014) and DSM-V (American Psychiatric Association 2013).

The neuropsychological assessment took place in the Greek Association of Alzheimer’s Disease and Related Disorders and included the following tools: the Geriatric Depression Scale (Fountoulakis et al. 1999; Yesavage et al. 1982), the Beck Depression Inventory (Beck et al. 1961), the Beck Anxiety Inventory (Beck et al. 1988), and the Short Anxiety Screening (Sinoff et al. 1999; Grammatikopoulos et al. 2010); these were used to exclude affective disorders. In addition, the Neuropsychiatric Inventory (Politis et al. 2004; Cummings et al. 1994) was also used to exclude neuropsychiatric symptoms. The Mini-Mental State Examination (Fountoulakis et al. 2000; Folstein et al. 1975) and the Montreal Cognitive Assessment (Poptsi et al. 2019; Nasreddine et al. 2005) were used to screen general cognitive status, and the Functional Cognitive Assessment (Kounti et al. 2006) was used to assess executive functions in six daily activities. Furthermore, additional standardized cognitive tests were applied to assess memory, attention, executive functions, and language abilities. The Global Deterioration Scale (GDS, Reisberg et al. 1982) was used to assess participants’ status in terms of deterioration progression. Therefore, based on the GDS, individuals with no cognitive decline and normal functioning—exhibiting no impairments—were classified as stage 1. In contrast, individuals with mild cognitive impairment (MCI) were assigned to stage 3. A detailed presentation of all the applied neuropsychological tests can be found in the study of Tsolaki et al. (2017). 

Exclusion criteria for both groups were as follows: (a) history of psychiatric disorder; (b) substance abuse or alcoholism; (c) history of traumatic brain injury; (d) history of neurological disorders (brain tumor, epilepsy, encephalitis, Parkinson’s disease, multiple sclerosis); (e) diabetes (types I and II); (f) cardiovascular diseases; (g) sensorimotor deficits that could interfere with study procedures; and (h) vitamin B12 deficiency; for the HC group, presence of subjective cognitive complaints was also an exclusion criterion. 

Univariate analysis of variance (ANOVA) was conducted to examine whether the two groups differed in age (in years) and years of education. The statistical analysis revealed no significant differences between the two groups for age, F(1, 98) = 1.56, *p* = .215, or for years of education, F(1, 98) = 1.60, *p* = .209. In addition, chi-square analysis regarding gender and group showed that there were also no statistically significant differences between the groups, χ^2^ (1) = 0.05, *p* = .832. Hence, the two groups were matched in age, education, and gender distribution (see Table 1). 

### 2.3. Procedure

Participants were recruited from the “Agia Eleni” daycare center of the Greek Association of Alzheimer’s Disease and Related Disorders and through the Aristotle University of Thessaloniki, with assistance from undergraduate psychology students completing clinical internships. If participants met this study’s inclusion criteria, they were asked if they would like to volunteer for this study. Those who agreed were informed that this study’s neuropsychologist would review their eligibility and, if deemed suitable, would contact them. During the initial communication, the neuropsychologist provided information on this study’s purpose and procedures, explaining that participants would need to schedule two morning appointments at their convenience to complete some tests. The testing procedure was divided into two appointments, each lasting a maximum of one hour, to minimize the potential interference of fatigue with the tests. The test sequence was counterbalanced. Both sessions were scheduled to take place within a one-week interval. At the beginning of the first appointment, the participants were provided with written informed consent forms that outlined this study’s objectives and assured them of the confidentiality of their personal information. Participants were not reimbursed for their participation.

### 2.4. Cognitive Measures

We selected an executive functions task and an episodic memory task to assess cognition, as these are the primary cognitive domains affected by aMCI. Additionally, both tasks were suitable for incorporating the metacognitive measures (described in Section 2.5).

#### 2.4.1. Wisconsin Card Sorting Test—64 Card Version (WCST-64) 

The WCST-64 (Kongs et al. 2000) is a shortened version of the original (Berg 1948; Grant and Berg 1948), and it consists of 64 sorting cards, as opposed to the original 128 cards, of different colors, shapes, and numbers. The test measures cognitive flexibility, cognitive set-shifting, and the ability to use feedback to guide problem-solving. Despite its reduced length, the WCST-64 retains the original test’s core structure and administrative procedures, requiring participants to match cards according to undisclosed, shifting rules while receiving feedback to guide their responses. The WCST-64 retains solid psychometric properties, including substantial test–retest reliability (Greve et al. 2002; Chiu and Lee 2019; Axelrod et al. 1992) and construct validity, as shown by its capacity to detect frontal lobe dysfunction (Nyhus and Barceló 2009) and its associations with other measures of executive functioning (Miyake et al. 2000). The WCST-64 provides a time-saving alternative to the full-length WCST while maintaining its diagnostic value and adaptability across various populations and clinical environments (Axelrod 2002). 

The test measures several cognitive scores that provide insights into an individual’s performance (Heaton et al. 1993). Some key scores derived from WCST include the following: (1) Total correct: This score represents the total number of correct responses the participant gave throughout the test. A higher score indicates better performance and cognitive flexibility. (2) Total errors: This score represents the total number of incorrect responses the participant gave during the test. A lower score indicates better performance and fewer mistakes made. (3) Perseverative responses: This score represents the number of times the participant continued to use a previously correct sorting rule, even after it was no longer valid. A lower score indicates better cognitive flexibility and adaptability to changing rules. (4) Perseverative errors: This score represents the number of errors made by the participant due to the persistent application of an incorrect rule or strategy, even after receiving feedback that it was no longer valid. A lower score indicates better cognitive flexibility and ability to adapt to new information. (5) Nonperseverative errors: This score represents the number of incorrect responses that were not perseverative. A lower score indicates better performance in terms of adaptability and problem-solving. (6) Categories completed: This score represents the number of categories (out of a possible six) that the participant successfully completed during the test. A higher score indicates better cognitive flexibility and abstract reasoning. (7) Trials to complete first category: This score represents the number of trials needed for the participant to complete the first category effectively. A lower score indicates a quicker understanding of the sorting rules and more efficient problem-solving skills. (8) Failure to maintain set: This score represents the number of times the participant failed to maintain a correct sorting rule after successfully applying it for a few consecutive trials. A lower score indicates better cognitive stability and consistency in applying learned rules.

#### 2.4.2. Doors and People 

The Doors and People (Baddeley et al. 1994) is a tool developed to evaluate memory function, and it is divided into four parts, each of which assesses different aspects of memory: people, doors, figures, and names. The test has been adapted and validated for the Greek population (Arabatzi and Masoura 2012). Ιt is a reliable tool with ecological validity and satisfactory internal validity (Cronbach’s α = 0.80).

The people subtest measures immediate (three trials) and delayed verbal recall by presenting a list of names and later asking the participant to recall them. Specifically, the stimuli comprise photos of four characters, with their names and professions displayed underneath. Each image is shown for 3 s while the character’s name and occupation are read aloud (e.g., This is a doctor. His name is Hλίας Τσακίρης (Elias Tsakiris)). This process is repeated until all four names are accurately remembered (with a maximum of three attempts). Participants are asked to recall this information immediately following the presentation and after a 5–10 min interval. One point is given for each correct first and last name plus an extra point for each proper pairing. The total score is calculated by summing the individual scores from each trial (score range: 0–36) (Hess and D’Amato 1999). 

The doors subtest assesses visual recognition by showing pictures of doors and later asking the participant to identify the previously seen doors among new ones. Specifically, participants are shown 24 door images divided into two sets (an easy set and a challenging set). After the presentation, they must select the previously displayed door from four options (three distractors and the target door). In the first set (Part A), the distractors are different types of doors compared to the target door (e.g., a garage door, a German door, a front door), while in the second set (Part B), the distractors are of the same door category (e.g., all stable doors). One point is given for each correct answer, and the total score is derived from the sum of the scores in each set (score range: 0–24) (Hess and D’Amato 1999). 

The figures subtest measures immediate and delayed visual recall by showing a set of figures and later asking the participant to draw as many as they can remember. Participants are shown four-line drawings of crosses and asked to reproduce them immediately following the presentation and after a 5–10 min interval. The shapes are displayed until the participant can correctly recall them (with a maximum of three attempts). Each accurately drawn shape earns three points, and the total score is calculated by summing the individual scores from each trial (score range: 0–36) (Hess and D’Amato 1999). 

Finally, the names subtest assesses verbal recognition by presenting a list of names and later asking the participant to identify which names were previously presented. Participants are shown twenty-four names (including both first and last names), divided into two sets (an easy set and a challenging set), each presented for 3 s, and they are asked to read them aloud. Following the presentation, participants must select the previously displayed name from four options (three distractors and the target name). The second set (Part B) features names where distractors are more like the target name. One point is awarded for each correct answer, and the total score is obtained by summing the scores from each set (score range: 0–24) (Hess and D’Amato 1999). 

### 2.5. Metacognitive Measures

For the present study, two cognitive tests—the Wisconsin Card Sorting Test and the Doors and People test—were applied using a metacognitive version (Koren et al. 2004; Koriat and Goldsmith 1996). After each response, participants had to provide answers to the following questions: (1) “What is your degree of confidence in the correctness of this answer?” (reflecting FOC estimations); and (2) “Would you like your response to be included in the total score?” (reflecting metacognitive control as a process to decide whether to volunteer or not a response to maximize final score performance). The response to the first question was given on a 4-point Likert scale (1 = not at all certain, 4 = totally certain), while the response to the second question was given in yes/no format. This was under the premise that a correct “yes” would earn them a point, an incorrect “yes” would deduct a point, and a “no” response, irrespective of its correctness, would not alter their score. 

Based on the responses to these two metacognitive questions, four metacognitive variables were calculated: (1) mean item-by-item confidence ratings (1–4 range); (2) accuracy score (as the ratio of the correct volunteered responses, i.e., correct yes, to the total volunteered responses, i.e., total yes. It reflects the extent to which one’s responses can be trusted and relies on monitoring and control processes); (3) global monitoring (which refers to an individual’s ability to assess their overall knowledge or performance of a task. It is measured as the difference between the total number of the correct responses, i.e., objective performance, and the total number of the volunteered responses, i.e., total yes. Values below zero indicate overconfidence, while values above zero indicate underconfidence); and (4) wrong yes (the number of incorrect volunteered responses, where lower values suggest a more cautious decision-making approach and higher values indicate a riskier one).

#### 2.5.1. Metacognitive Ability

To evaluate metacognitive ability (either overconfidence or underconfidence), the ratio of relative confidence to cognitive performance was estimated using the following calibrating formula: Metacognitive Ability = Relative Confidence/Cognitive Score.

Relative confidence represents the mean item-by-item confidence and ranges between 1 (lowest confidence) and 4 (highest confidence). The cognitive score represents the accuracy in performance calculated as the ratio of correct responses to total test items, resulting in a range from 0 (no correct answers) to 1 (all answers are correct). Thus, a score of 4 denotes perfect alignment. This means the participant is highly confident (a rating of 4) and performs perfectly (cognitive score of 1). Consequently, scores below 4 indicate underconfidence (i.e., if a participant has a relative confidence score of 2 (somewhat confident) but a high cognitive score of 0.80, the calibration score would be 2.5). In contrast, scores above 4 indicate overconfidence (i.e., if a participant’s relative confidence is 4 (very high) but the participant achieve a cognitive score of only 0.5, their metacognitive calibration would be 8).

#### 2.5.2. Brier Score (Brier 1950)

The Brier score was calculated using Microsoft Excel (version 16.76) to quantify participants’ ability to discriminate between correct and incorrect answers based on their yes/no responses (forecast) and their actual performance of correct/wrong responses (actual event). This score is suitable for binary outcomes and captures the mean squared difference between predicted probabilities and actual results. It was computed for each test and subtest. A score of 0 indicates a perfect forecast, while a score of 1 indicates the least accurate forecast.

### 2.6. Statistical Analysis

The statistical analysis was conducted using IBM SPSS Statistics Version 27 (IBM Corp. Released 2020. IBM SPSS Statistics for Macintosh, Version 27.0. Armonk, NY, USA: IBM Corp.). To test whether the two groups differed in performance as well as in metacognitive measures, the following analyses were conducted: (a) multivariate analysis of variance (MANOVA); and (b) one-way ANOVA. Partial eta-squared (η^2^) was used to estimate the effect size. A *p*-value < 0.05 was considered indicative of statistical significance.

### 2.7. Ethics 

Participants were informed about this study’s purpose orally and in writing and assured of data confidentiality. They provided written consent, acknowledging voluntary participation with the option to withdraw at any time. Demographic data, including age, gender, and education, were collected in compliance with the European Union law since 28 May 2018, which permits the use of sensitive personal data for research purposes. Participants were informed and agreed that their data could be removed from the web database upon written request. The research protocol received approval from the Scientific and Ethics Committee of the Greek Association of Alzheimer’s Disease and Related Disorders (Approval Code: 29/15-02-2017), adhering to the guidelines outlined in the Declaration of Helsinki.

## 3. Results

### 3.1. Cognitive Performance: Group Differences in WCST and Doors and People Tests

The sums of correct responses for each participant on each subtest of the Doors and People test were calculated, and similarly, the scores for the WCST were computed. MANOVA was conducted to investigate the differences in performance between the two groups on the WCST and the Doors and People subtests. As dependent variables were identified, eight cognitive scores for the WCST (total correct, total errors, perseverative responses, perseverative errors, nonperseverative errors, categories completed, trial to complete first category, and failure to maintain category) and six variables for the Doors and People subtests (people, doors, figures, names, verbal loss (people immediate recall score—people delayed recall score), and visual loss (figures immediate recall score—figures delayed recall score)), and group was identified as the independent variable.

#### 3.1.1. Wisconsin Card Sorting Test

The analysis showed that the aMCI group generally performed worse in the WCST compared to the HC group, F(14, 83) = 6.06, *p* < .001. Specifically, the aMCI group had fewer correct responses and completed fewer categories compared to the HC group. Additionally, the aMCI group made more errors and required more trials to complete the first category (see Table 2 for more details).

#### 3.1.2. Doors and People

Similarly, the aMCI group performed worse in the Doors and People test than the HC group, F(14, 83) = 6.06, *p* < .001. Specifically, the aMCI group performed worse in the recall subtests (verbal and visual; people and figures, respectively) and in the recognition subtests (visual and verbal; doors and names, respectively). Interestingly, the two groups were equally able to retain the learned verbal and visual information since no significant differences between the two groups were detected for verbal loss or visual loss scores (see Table 2 for more details).

### 3.2. Group Differences in Metacognitive Monitoring

#### 3.2.1. Feeling of Confidence

One-way ANOVAs were conducted to test whether the two groups differed regarding their mean feeling of confidence across the tasks. Feeling of confidence was identified as the dependent variable, and “group” was identified as the independent variable. This analysis was carried out separately for each test (or subtest). Based on these results, the aMCI group reported statistically less confidence compared to the HC group across all tasks, indicating some level of awareness of the aMCI group regarding their difficulties while performing the tasks. Additionally, the variance in confidence levels within the aMCI group was more spread out, indicating a broader range of confidence among these participants compared to the other group. For a detailed description of the results, see Table 3.

#### 3.2.2. Metacognitive Ability

Metacognitive ability was calculated for each test separately, yielding seven distinct ratios corresponding to WCST, people (immediate), doors, people (delayed), figures (immediate), names, and figures (delayed). A MANOVA analysis was conducted to test group differences, with the seven scores for metacognitive ability identified as dependent variables and the group as the independent factor. Results revealed a significant group effect on metacognitive ability, F (7, 82) = 4.61, *p* < .001. Specifically, participants with aMCI exhibited overconfidence (with scores exceeding 4), indicating a discrepancy between their reported confidence and actual performance compared to the HC group. Significant differences between the two groups were detected for all the Doors and People subtests except names (verbal recognition). While no significant differences in metacognitive ability were observed for names (verbal recognition), the aMCI group showed a minor overestimation with a mean score of 4.27 (SD = 0.88) compared to the perfect calibration score of 4. In contrast, the HC group had a mean score of 4.24 (SD = 0.51). For the WCST, the observed overconfidence for both groups was more pronounced: the aMCI group had a mean of 4.79 (SD = 0.58) and the HC group had a mean of 4.66 (SD = 0.58). For a detailed description of the results, see Table 4.

### 3.3. Group Differences in Metacognitive Control

#### 3.3.1. Monitoring Accuracy, Global Monitoring, and “Wrong Yes”

One-way ANOVAs were conducted to test whether the two groups differed regarding their monitoring accuracy, global monitoring, and wrong yes (metacognitive control) across the tasks. The online metacognitive measures were treated as dependent variables, while “group” was identified as the independent variable. This examination was carried out individually for each test (or subtest). The findings showed statistically significant differences between the two groups in relation to all three indexes of metacognitive control. Specifically, the aMCI group showcased poorer monitoring accuracy and global monitoring than the HC group, and they included more incorrect responses in their final score than the HC group. For a detailed description of the results, see Table 5.

#### 3.3.2. Group Differences in Yes/No Accuracy Discrimination

To directly assess the accuracy of yes/no decisions and actual performance item by item, the Brier score was computed for each test and subtest, resulting in seven Brier scores corresponding to WCST, people (immediate), doors, people (delayed), figures (immediate), names, and figures (delayed). A MANOVA analysis was conducted to test group differences, with the seven Brier scores identified as dependent variables and the group as the independent factor. The results underscore the difficulties aMCI participants had in distinguishing between correct and incorrect responses compared to the HC group, as evidenced by a significant main effect of the group on probabilistic accuracy, F(7, 92) = 6.40, *p* < .001. As illustrated in Table 6, the aMCI group was statistically less likely to volunteer a correct response across all tasks when compared to the HC group. 

Overall, the results indicated significant differences between the two groups in all metacognitive measures, with the aMCI group exhibiting poorer metacognition in both monitoring and control. Notably, the aMCI group consistently demonstrated greater variance across all variables, as evidenced by higher standard deviations, suggesting a more diverse range of responses compared to the HC group.

## 4. Discussion

In the present study, we sought to examine metacognition in individuals with aMCI and compare their performance with healthy controls in two different cognitive tasks, utilizing online measures of metacognition. Our findings contribute to the growing body of knowledge on metacognition in MCI. Below, we discuss the results in light of the objectives and hypotheses, the implications of the findings, and potential directions for future research.

**Hypothesis** **1.** *Participants with aMCI will perform worse than cognitively healthy (HC) older adults in both cognitive tasks, indicating inferior cognitive performance in the aMCI group.*

As anticipated, the two groups showed differences in cognitive performance, with the aMCI group performing worse in both tasks compared to the HC group. These findings are consistent with the previous literature and the diagnosis of aMCI. Individuals with aMCI, and specifically those with multidomain deficits, exhibit impairments in several cognitive areas, including episodic memory, working memory, prospective memory, verbal fluency, and executive functions, such as control and cognitive flexibility (Chehrehnegar et al. 2020; Ávila et al. 2015; Rattanavichit et al. 2022). According to our findings, aMCI individuals underperformed compared to HC in tasks requiring visual and verbal recognition and recall. Interestingly, no significant differences were detected for verbal and visual loss. This could be attributed to the simplicity of the tasks; they required a short time commitment of 5 to 10 min to memorize just four items. This setting likely allowed aMCI individuals to perform at their highest level of capacity (Arabatzi and Masoura 2012), enabling them to retrieve the learned items effectively. 

Regarding performance in WCST, aMCI individuals performed worse, as reflected by most of their WCST scores compared to the HC participants, underlining deficits in cognitive flexibility and problem-solving skills. No differences were observed regarding “trials to complete the first category” and “failure to maintain category”, echoing prior research that suggests these criteria may not effectively discriminate between healthy older adults and those with MCI (for review, see Guarino et al. 2020). However, the absence of differences in “perseverative errors” was unexpected, given its reflection on cognitive flexibility. Possibly, the WCST might not be as sensitive as tasks like the computerized Stroop, Flanker, Go/No Go, or Trail Making Test, Part B in detecting MCI-related cognitive flexibility deficits, as recent reviews suggest (for reviews, see Guarino et al. 2020; Miles et al. 2021). Despite this, the other scores from the WCST did show cognitive flexibility deficits in the aMCI group, aligning with available findings indicating deficits in cognitive flexibility in MCI, both amnestic and nonamnestic (Corbo and Casagrande 2022; Gonçalves et al. 2019; Rattanavichit et al. 2022; Ávila et al. 2015).

**Hypothesis** **2.** *Individuals with aMCI would express lower confidence levels than HC individuals (Hypothesis 2a), but they would show poorer calibration in relation to their performance (Hypothesis 2b).*

In line with Hypothesis 2a, the aMCI group reported significantly lower confidence ratings in comparison to the HC group for all tasks, meaning that participants in the aMCI group were aware of their cognitive struggles while performing the tasks. These results agree with studies indicating a preserved metacognitive awareness in individuals with MCI (Seelye et al. 2010; Clare et al. 2013; Chudoba and Schmitter-Edgecombe 2020). Specifically, the studies showed that, following exposure to task-related experiences, individuals with MCI were capable of adjusting their predictions about their performance. 

However, even though individuals with aMCI had lower relative confidence compared to the HC group, the relationship between their relative confidence and actual performance indicated overconfidence, confirming Hypothesis 2b. This result aligns with previous studies showing deficits in metacognitive monitoring skills. Specifically, although individuals with MCI might acknowledge their cognitive difficulties and consistently report lower confidence levels in their cognitive performance, their evaluations lack accuracy (Ryals et al. 2019; Pennington et al. 2021; Anderson and Schmitter-Edgecombe 2010; Perrotin et al. 2007; Chi et al. 2022). In other words, their subjective assessments do not always align with their actual performance; thus, while individuals with MCI appear to be aware of their cognitive struggles to some extent, evidenced by their reported lower confidence, their ability to accurately gauge their cognitive performance was disrupted, as reflected by poorer calibration.

These findings suggest a nuanced view of metacognition in MCI, with some aspects being relatively preserved while others are affected. This adds a layer of complexity to our understanding of metacognitive function in the context of MCI.

**Hypothesis** **3.** *Participants in the aMCI group would display reduced precision in their decisions to volunteer correct or incorrect responses compared to the participants in the HC group.*

As anticipated, participants in the aMCI group were less accurate than those in the HC group when deciding which responses to include in their score. This was evident across all three measures of metacognitive control and the direct relationship as determined by the Brier score between yes/no decisions and actual performance. It is interesting to note, though, that in most tasks, mean monitoring accuracy for the aMCI group ranged from 0.70 to 0.88. This indicates that 70% to 80% (a satisfactory percent of accuracy) of the aMCI group’s responses can be considered reliable, indicating some degree of monitoring accuracy. Investigating whether it could be improved via specific cognitive training programs targeting metacognitive skills would be interesting. Nevertheless, in almost all tasks (except DnP—names), the aMCI group showed overconfidence with a more considerable discrepancy between volunteered responses and actual performance than the HC group, as reflected by the global monitoring variable, and opted to include more incorrect responses in their final score.

Consequently, despite the aMCI group’s lower confidence, they did not adopt a more conservative decision-making process. Instead, they appeared more willing to volunteer responses. This could be attributed to deficits in metacognitive accuracy, which affect their ability to monitor and regulate cognitive performance effectively. Their lower confidence might reflect a lower self-efficacy, formed from their metacognitive knowledge and beliefs about their cognitive abilities and shaped by everyday cognitive challenges.

Evidence from neuroimaging studies offers further insight into metacognitive aging. In their recent review, Fleur et al. (2021) presented a comprehensive overview of the neural structures implicated in metacognition, aligning with Vaccaro and Fleming’s (2018) meta-analysis. They highlighted the key role of the precuneus, parahippocampal gyrus, insula, and regions of the prefrontal cortex (PFC)—including its anterior and lateral areas—in underpinning self-reflective processes and metacognitive judgments and feelings. The anterior cingulate cortex (ACC) along with the medial and dorsal regions of the PFC were identified as crucial for metacognitive control and regulation. Research has further highlighted the role of the anterior PFC in metacognitive monitoring accuracy, with studies specifically suggesting that prospective judgments (JOLs and FOKs) are subserved by the medial PFC and retrospective judgments (FOCs) by the lateral PFC and anterior regions of the PFC (Baird et al. 2013; Fleming and Dolan 2012; Chua et al. 2014). In addition, a recent study underscored the crucial function of the lateral PFC in metacognition, identifying it as a central neural hub engaged in both metacognitive monitoring and control processes (Boldt and Gilbert 2022). The PFC areas undoubtedly form the fundamental neural basis of metacognition, and studies have demonstrated functional abnormalities within the FPN in MCI (Li et al. 2015; Sheng et al. 2017; Terry et al. 2015; Zhang et al. 2015; Zhao et al. 2022). Other essential areas for metacognition, such as the precuneus and hippocampus, also exhibit structural and functional changes in MCI (Jin et al. 2012; Traschütz et al. 2020; Korf et al. 2004; Sexton et al. 2010; Haussmann et al. 2017; Csukly et al. 2016). The medial PFC and precuneus, which are integral components of the DMN, have been highlighted in the context of MCI (Eyler et al. 2019; Terry et al. 2015; Li et al. 2015). Specifically, the DMN exhibits abnormal activity during cognitive tasks and disrupted connectivity in MCI (Fox et al. 2005). Consequently, these neuroimaging findings lend further credence to the effects of MCI on metacognition, underscoring the necessity for ongoing research in this field.

It is essential to acknowledge the limitations of the current study. Recent studies have introduced more sensitive methods to assess metacognitive efficiency, such as signal detection theory and meta-d’ (type 2 signal detection). These approaches typically rely on computerized tasks with specific structures, while our study utilized two paper-and-pencil neuropsychological tasks. Despite this limitation, our findings hold significance as they propose a potential method for integrating metacognitive assessment into neuropsychological evaluations. It is worth noting that our sample primarily consisted of highly educated individuals, which could impact their cognitive and metacognitive abilities. Therefore, the generalizability of the findings to a more diverse population with varying education levels may be limited. Furthermore, the aMCI participants did not exhibit severe cognitive deterioration and were in the early phase of the MCI continuum. As a result, the differences between the aMCI and healthy control groups might be less pronounced than if the study had included individuals with more severe cognitive deficits (demented).

Future research should focus on investigating metacognitive processes in MCI using more sensitive assessment methods combined with neuroimaging data. Including participants with a broad range of education levels and severity of cognitive deficits is also essential. Longitudinal studies and comparisons between various MCI subtypes may provide valuable insights into the role of metacognition in cognitive decline. Developing and implementing metacognitive training programs tailored to the specific needs of individuals with MCI could offer promising interventions, addressing cognitive impairment and enhancing overall cognitive performance. Incorporating metacognitive assessment into neuropsychological evaluations may allow clinicians to adopt a comprehensive approach when designing and implementing interventions for aMCI populations, ultimately improving their quality of life.

## 5. Conclusions

The findings highlight the complex interplay between metacognitive monitoring, metacognitive control, and cognitive performance in those with aMCI. Although these participants demonstrated some level of cognizance about their cognitive performance, as indicated by their confidence levels, they were overconfident in relation to their actual performance. Furthermore, participants with aMCI faced difficulties distinguishing right and wrong answers, a deficit clearly manifested in their choices of which answers to volunteer. These findings highlight deficiencies in both metacognitive monitoring—the ability to assess one’s performance—and in metacognitive control—the ability to manage and steer cognitive processes effectively, a fact that is corroborated by the existing neuroimaging data on MCI. In essence, people with aMCI appear aware that something is off but struggle to pinpoint the issue or how to address it accurately. This raises further questions about the psychological impact of this awareness on aspects like distress and depression or how it influences coping behaviors in cognitively demanding situations (Beaudoin 2018; Cherry et al. 2019), such as the implementation of effective control mechanisms like efficient allocation of study time (Froger et al. 2011) or the utilization of cognitive strategies (Tomaszewski Farias et al. 2018). Finally, it is important to note that these deficits were observed across both tasks, implying that metacognitive deficits in aMCI are not confined to specific tasks but rather are a general issue aligning with the domain-generality approach for metacognitive aging (McWilliams et al. 2023). Indeed, these findings warrant further investigation, as it is crucial to understand the potential consequences of such deficiencies in real-life situations in which older adults make critical decisions related to healthcare, retirement, and financial planning.

## Figures and Tables

**Table 1:** Participants’ demographic characteristics.

	aMCI	HC		
	Mean (SD)	Mean (SD)	F	*p*
Age	62.76 (6.67)	61.20 (5.78)	1.56	n.s. ^1^
Education	14.58 (2.87)	15.32 (2.99)	1.60	n.s.
Gender (f/m)	34/16	33/17	χ^2^	n.s.

^1^ n.s. = nonsignificant difference between the two groups.

**Table 2:** Group differences in cognitive scores.

	aMCI	HC			
	Mean (SD)	Mean (SD)	F	*p* *	η^2^
Total correct	43.76 (10.35)	49.76 (5.95)	12.38	.001	0.114
Total errors	19.94 (9.95)	13.98 (6.03)	12.85	.001	0.118
Perseverative responses	1.78 (1.62)	2.51 (1.93)	4.17	.044	0.042
Perseverative errors	8.88 (4.54)	8.12 (4.24)	0.72	n.s. ^1^	0.007
Nonperseverative errors	11.06 (7.44)	5.88 (3.19)	20.09	.001	0.173
Categories completed	2.80 (1.63)	3.65 (1.11)	9.24	.003	0.088
Trials to complete 1st category	15.71 (10.93)	13.63 (5.42)	1.43	n.s.	0.015
Failure to maintain category	0.45 (0.84)	0.55 (0.87)	0.35	n.s.	0.004
People	20.18 (7.15)	27.45 (4.59)	35.85	.001	0.272
Doors	15.49 (2.92)	18.16 (1.88)	29.04	.001	0.232
Figures	32.67 (4.68)	34.90 (1.77)	9.67	.002	0.092
Names	17.39 (3.03)	19.47 (2.60)	13.31	.001	0.122
Verbal loss	−1.53 (1.78)	−0.94 (1.23)	3.66	n.s.	0.037
Visual loss	−0.14 (0.91)	−0.08 (0.34)	0.19	n.s.	0.002

* *p* < .05. ^1^ n.s. = nonsignificant difference between the two groups.

**Table 3:** Group differences in feeling of confidence.

	aMCI	HC		
	Mean (SD)	Mean (SD)	F	*p*
Wisconsin Card Sorting Test	3.28 (0.63)	3.56 (0.45)	6.29	.014
DnP—people (immediate verbal recall)	2.72 (0.59)	3.11 (0.43)	14.60	<.001
DnP—doors (visual recognition)	2.91 (0.52)	3.10 (0.36)	4.10	.046
DnP—people (delayed recall)	3.15 (0.90)	3.74 (0.38)	17.96	<.001
DnP—figures (immediate visual recall)	3.69 (0.49)	3.90 (0.16)	8.38	.005
DnP—names (verbal recognition)	3 (0.47)	3.43 (0.31)	20.60	<.001
DnP—figures II (delayed verbal recall)	3.77 (0.44)	4 (0.04)	13.31	<.001

**Table 4:** Group differences in metacognitive ability.

	aMCI	HC		
	Mean (SD)	Mean (SD)	F	*p*
Wisconsin Card Sorting Test	4.79 (0.83)	4.66 (0.58)	0.84	n.s. ^1^
DnP—people (immediate verbal recall)	6.35 (2.41)	4.59 (0.93)	22.42	<.001
DnP—doors (visual recognition)	4.59 (0.86)	4.10 (0.49)	11.36	.001
DnP—people (delayed recall)	5.70 (2.41)	4.55 (1.06)	8.92	.004
DnP—figures (immediate visual recall)	4.67 (1.28)	4.20 (0.43)	5.70	.019
DnP—names (verbal recognition)	4.27 (0.88)	4.24 (0.51)	0.05	n.s.
DnP—figures II (delayed verbal recall)	4.63 (1.74)	4.10 (0.60)	3.97	.049

^1^ n.s. = nonsignificant difference between the two groups.

**Table 5:** Group differences in monitoring accuracy, global monitoring, and “Wrong Yes”.

	aMCI	HC		
	Mean (SD)	Mean (SD)	F	*p*
Monitoring Accuracy ^a^				
Wisconsin Card Sorting Test	0.70 (0.14)	0.79 (0.11)	10.90	.001
DnP—people (immediate verbal recall)	0.57 (0.22)	0.82 (0.15)	43.77	<.001
DnP—doors (visual recognition)	0.73 (0.15)	0.84 (0.10)	16.20	<.001
DnP—people (delayed recall)	0.71 (0.28)	0.91 (0.14)	20.37	<.001
DnP—figures (immediate visual recall)	0.86 (0.21)	0.95 (0.10)	8.76	.004
DnP—names (verbal recognition)	0.77 (0.14)	0.85 (0.12)	11.82	<.001
DnP—figures II (delayed verbal recall)	0.84 (0.26)	0.97 (0.16)	8.93	.004
Global Monitoring ^b^				
Wisconsin Card Sorting Test	−15.31 (10.28)	−11.32 (8.16)	4.58	.035
DnP—people (immediate verbal recall)	−3.92 (2.45)	−1.76 (1.74)	25.64	<.001
DnP—doors (visual recognition)	−3.54 (5.73)	−1.10 (3.96)	6.15	.015
DnP—people (delayed recall)	−0.98 (0.98)	−0.34 (0.52)	16.67	<.001
DnP—figures (immediate visual recall)	−1.48 (2.36)	−0.50 (1.18)	6.82	.010
DnP—names (verbal recognition)	−2.76 (4.64)	−1.74 (3.85)	1.43	n.s.
DnP—figures II (delayed verbal recall)	−0.60 (0.97)	−0.14 (0.64)	7.85	.006
Wrong Yes ^c^				
Wisconsin Card Sorting Test	17.10 (8.58)	13 (7.18)	6.67	.011
DnP—people (immediate verbal recall)	4 (2.38)	1.86 (1.58)	28.05	<.001
DnP—doors (visual recognition)	5.52 (3.63)	3.34 (2.39)	12.55	<.001
DnP—people (delayed recall)	0.98 (0.98)	0.32 (0.51)	17.83	<.001
DnP—figures (immediate visual recall)	1.60 (2.27)	0.54 (1.15)	8.70	.004
DnP—names (verbal recognition)	4.7 (3.02)	3.18 (2.69)	7.06	.009
DnP—figures II (delayed verbal recall)	0.62 (0.97)	0.14 (0.63)	8.58	.004

Notes: ^a^ Monitoring accuracy = correct volunteered responses out of total volunteered responses. ^b^ Global monitoring = total volunteered responses − actual correct responses. ^c^ Wrong yes = total wrong volunteered responses.

**Table 6:** Group differences in Brier scores.

	aMCI	HC		
	Mean (SD)	Mean (SD)	F	*p*
Wisconsin Card Sorting Test	0.29 (0.13)	0.22 (0.10)	7.20	.009
DnP—people (immediate verbal recall)	0.35 (0.25)	0.15 (0.11)	26.96	<.001
DnP—doors (visual recognition)	0.31 (0.16)	0.24 (0.10)	11.86	<.001
DnP—people (delayed recall)	0.28 (0.29)	0.08 (0.12)	20.91	<.001
DnP—figures (immediate visual recall)	0.15 (0.20)	0.05 (0.11)	10.21	.002
DnP—names (verbal recognition)	0.27 (0.12)	0.20 (0.12)	9.49	.003
DnP—figures II (delayed verbal recall)	0.15 (0.23)	0.01 (0.08)	15.26	<.001

## Data Availability

The data of this study are available at DOI: 10.17632/wsk4xd5m62.2.
